# Association of estimated pulse wave velocity with outcomes following drug-coated balloon therapy in elderly coronary artery disease patients

**DOI:** 10.3389/fcvm.2026.1706461

**Published:** 2026-02-13

**Authors:** Fengyun Zhang, Jianfan Shen, Lin Bo, Wei Bao

**Affiliations:** 1 Department of Cardiology, Affiliated Hospital of Xuzhou Medical University, Xuzhou, Jiangsu, China; 2 Department of Critical Care Medicine, Affiliated Hospital of Xuzhou Medical University, Xuzhou, Jiangsu, China; 3 Department of Cardiology, Tongji Hospital, School of Medicine, Tongji University, Shanghai, China

**Keywords:** coronary heart disease, drug-coated balloon, elderly, major adverse cardiovascular events, target lesion revascularization

## Abstract

**Introduction:**

Drug-coated balloons (DCBs) constitute a vital therapeutic approach in the interventional management of coronary heart disease. Nevertheless, the risk factors for predicting target lesion revascularization (TLR) and major adverse cardiovascular events (MACE) specifically within the elderly population following DCB angioplasty remain incompletely understood. The study is to explore the relationship between estimated pulse wave velocity (ePWV) values and the risk of TLR and MACE in elderly patients undergoing DCB treatment, and to explore the optimal ePWV cutoff for clinical risk stratification.

**Methods:**

A total of 423 participants were stratified into quartiles based on their ePWV values. Baseline characteristics were compared among these quartiles. The associations between ePWV and the risk of TLR and MACE were evaluated using Cox regression models, adjusted for multiple covariates. Kaplan–Meier analysis with the log-rank test was utilized to assess survival differences. The optimal ePWV cutoff for risk stratification was identified through maximally selected rank statistics. Subgroup analyses were performed to examine interactions between ePWV and clinical variables.

**Results:**

Differences emerged across ePWV quartiles for age, TLR, and MACE (all *P* < 0.05). Multivariate Cox regression revealed that elevated ePWV was associated with a higher risk of TLR (per unit increase: adjusted HR 1.46, 95% CI 1.18–1.79, *P* < 0.001) and MACE. A dose-response relationship was observed, with the highest ePWV quartile exhibiting the highest risk compared to the lowest. Kaplan–Meier curves showed differences in survival across quartiles (TLR: log-rank *P* = 0.012; MACE: *P* < 0.05). The optimal ePWV cutoff was identified at 10.91 m/s, differentiating high- and low-risk groups (log-rank *P* < 0.05). Notably, subgroup analysis revealed sex-based interactions for both TLR and MACE, with the predictive value being consistently more pronounced in females.

**Conclusion:**

Elevated ePWV was associated with a higher risk of TLR and MACE. An exploratory cutoff for ePWV at 10.91 m/s was identified, stratifying patients into distinct clinical risk groups.

## Introduction

Coronary artery disease (CAD) continues to be the primary cause of global mortality, with its impact intensified by aging populations and increasing rates of metabolic risk factors (1). Endovascular therapy, especially utilizing drug-coated balloons (DCBs), has transformed CAD management by lowering restenosis rates and enhancing long-term patency, particularly in complex lesions (e.g., small vessels, in-stent restenosis) (2–5). DCB technology facilitates effective revascularization and reduces the duration of dual antiplatelet therapy (4), which is especially crucial for elderly patients at high risk of bleeding, those with abnormal glucose metabolism, or those experiencing acute coronary syndrome.

Arterial stiffness, a critical mediator in CAD progression, reflects profound structural and functional alterations within the vascular wall, preceding the development of subclinical dysfunction (6). Furthermore, it can also serve as a predictive factor for in-stent restenosis (7, 8). Traditionally assessed via pulse wave velocity (PWV), the gold standard for gauging stiffness, its clinical application faces hurdles due to high costs and technical demands (9). Estimated PWV (ePWV), derived from readily accessible parameters like age and mean blood pressure, offers a low-cost, non-invasive alternative. It demonstrates a correlation with measured PWV and carries proven prognostic significance in CAD (10–12).

Recent studies demonstrate that ePWV stands out as a predictor of all-cause mortality in CAD patients, exhibiting a striking threshold effect. Once ePWV exceeds 11.15 m/s, the risk rises (10). Importantly, the success of DCB treatment is heavily contingent upon vascular physiology. Post-procedural hemodynamics, lesion calcification, and systemic vascular dysfunction are all established drivers of target lesion revascularization (TLR) and major adverse cardiovascular events (MACE) (13). Given that ePWV provides a direct measure of systemic arterial stiffness, a fundamental upstream driver of vascular dysfunction, it follows that ePWV is a promising predictor of responses to DCB treatment in CAD. Nevertheless, concrete evidence directly linking ePWV to DCB outcomes remains sparse.

This study examines and contextualizes the relationship between ePWV and DCB therapy outcomes in CAD. We explore the correlation between arterial stiffness, assessed by ePWV, and DCB treatment outcomes, while also highlighting critical research gaps and charting future directions.

## Methods

### Study population

**Figure 1:**
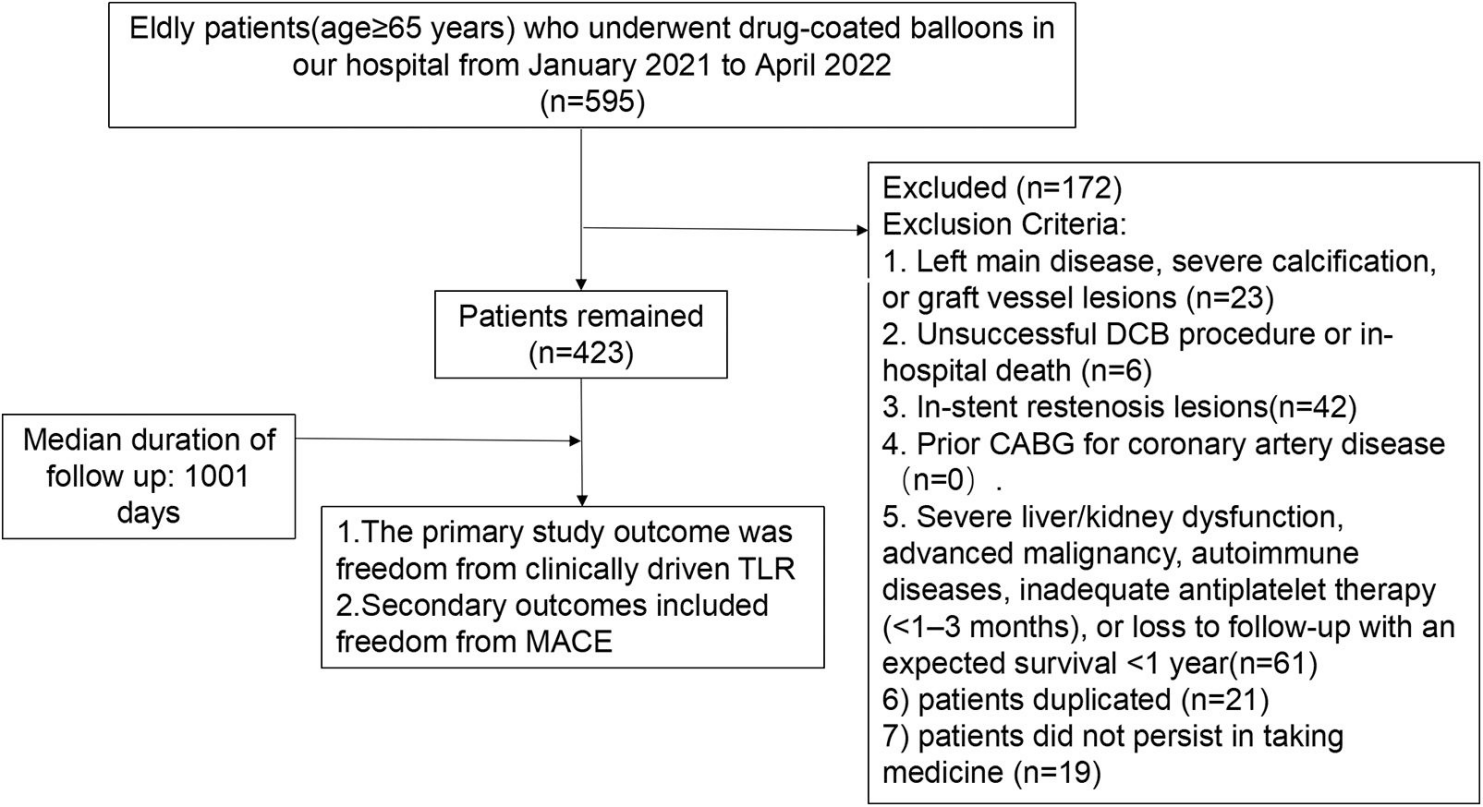
Flow chart of our study.

This retrospective observational study enrolled 423 elderly patients (aged ≥65 years) with *de novo* coronary artery lesions who underwent drug-coated balloon (DCB) treatment between January 2021 and June 2022. The inclusion criteria comprised meeting the indications for DCB treatment (14), achieving a TIMI flow grade of III post-angioplasty without type C or higher dissection, lesions amenable to complete coverage by a single DCB, and the availability of comprehensive clinical data. Key exclusion criteria included left main disease, severe calcification, or graft vessel lesions; an unsuccessful DCB procedure or in-hospital mortality; in-stent restenosis lesions; a history of CABG; severe liver/kidney dysfunction, advanced malignancy, autoimmune diseases, inadequate antiplatelet therapy (<1–3 months), loss to follow-up, or an expected survival of less than one year. The study protocol was approved by the Ethics Committee of the Affiliated Hospital of Xuzhou Medical University (Approval No.: XYFY2022-KL242-01). This study is an unregistered retrospective study and included a data availability statement. The flow chart for this study is shown in Figure 1.

### DCB intervention procedure

The lesion preparation process adhered to standardized protocols (13), which included pre-dilating with conventional balloons (at a balloon-to-artery ratio of 0.8-1.0. Cutting balloons were employed when necessary to minimize the risk of dissection. Paclitaxel-iopromide DCB angioplasty was performed using either a Jetsail Drug-Coated Balloon (MicroPort Medical, Shanghai, China) or a Sily Drug-Coated Balloon (Yinyi Biotech, Suzhou, China), inflated to the manufacturer's recommended pressure and maintained for 60 seconds. Procedural success was defined as achieving a TIMI flow grade III, with residual stenosis ≤30%, and no dissection. Bailout stenting was conducted if these criteria were not met. All patients received dual antiplatelet therapy (DAPT: aspirin + P2Y12 inhibitor) for a minimum of three months post-procedure, followed by single antiplatelet therapy. Systematically, guideline-recommended secondary prevention medications were adhered to.

### Calculation of ePWV

ePWV was calculated according to the formula validated for populations with major clinical risk factors, as established by the Reference Values for Arterial Stiffness Collaboration (15). Since all participants in this study were patients with coronary heart disease, the following equation was used (16, 17):

$$\begin{array}{rl} & \mathrm{ePWV}\;=\;9.587\;\text{-}\;0.402\;\times \;\mathrm{age}\;+\;4.561\;\times \;10^{\left(\text{-}3\right)}\;\times \;\mathrm{ag}e^{2}\;\text{-}\;2.621\\ & \;\;\times \;10^{\left(\text{-}5\right)}\;\times \;\mathrm{ag}e^{2}\;\times \;\mathrm{MBP}\;+\;3.176\;\times \;10^{\left(\text{-}3\right)}\;\times \;\mathrm{age}\\ & \;\times \;\mathrm{MBP}\;\text{-}\;1.832\;\times \;10^{\left(\text{-}2\right)}\;\times \;\mathrm{MBP} \end{array}$$

[where MBP = DBP + 0.4 × (SBP - DBP); SBP = systolic blood pressure, DBP = diastolic blood pressure].

### Follow-Up

Patients were followed at 1, 6, 12, 24, and 36 months post-procedure. Pre-procedure ePWV was measured at rest, repeated every six months in the first year, and annually thereafter. Standardized follow-ups were performed by trained researchers through outpatient visits or telephone interviews. The primary outcome was freedom from clinically driven target lesion revascularization (TLR), defined as reintervention within 5 mm of the original treated segment for >50% angiographic diameter stenosis with symptomatic worsening (18). Secondary outcomes included major adverse cardiovascular events (MACE), a composite of cardiac death, non-fatal acute myocardial infarction, and TLR. All events were adjudicated by investigators at participating centers.

### Data collection

Demographic, clinical, and laboratory data were collected, including the following demographics: age, sex, smoking status, hypertension, diabetes, and history of stroke or heart failure. Laboratory tests encompassed white blood cell count, C-reactive protein (CRP), mean corpuscular volume, red cell distribution width, alkaline phosphatase, fasting glucose, and lipid profile (total cholesterol, triglycerides, HDL-C, LDL-C). Angiographic data included the number of diseased vessels, lesion location, use of cutting balloon, pre-dilation pressure, and lesion diameter/length. Lesion complexity was quantified using the SYNTAX Score II 2020, retrospectively calculated using the online SYNTAX Score II calculator (19), incorporating key angiographic and clinical parameters. Assessments were performed independently by two cardiologists, with discrepancies resolved by consensus.

### Statistical analysis

Continuous variables are presented as mean ± standard deviation or median (interquartile range), and categorical variables as frequencies (percentages). Patients were stratified into quartiles based on ePWV values. Baseline characteristics were compared using ANOVA, Kruskal–Wallis, or chi-square tests, as appropriate. Associations between ePWV and risks of TLR and MACE were evaluated using unadjusted and multivariable-adjusted Cox proportional hazards models, with results expressed as hazard ratios (HR) and 95% confidence intervals (CI). The test for trend was performed by entering the quartile rank (1, 2, 3, and 4) as a continuous variable into the Cox regression models. Survival curves were plotted using the Kaplan–Meier method and compared with the log-rank test. The optimal ePWV cutoff for predicting TLR was identified by maximally selected rank statistics. Subgroup analyses were performed to examine interactions between ePWV and key clinical variables. All analyses were conducted using R version 4.3.3 and Zstats 1.0, with a two-sided p-value < 0.05 considered statistically significant.

## Results

### Baseline characteristics

A total of 423 participants were categorized into quartiles based on their ePWV values (Q1-Q4). During the follow-up period, 44 patients (10.40%) experienced target lesion revascularization (TLR), and 54 patients (12.77%) experienced major adverse cardiovascular events (MACE), which included 44 cases of TLR, 8 cases of cardiac death, and 2 cases of non-fatal acute myocardial infarction. Significant differences were observed across the quartiles for several key variables. Both systolic and diastolic blood pressure showed progressive increases from the first to the fourth quartile (*P* < 0.001), as did ePWV values and patient age (*P* < 0.001). The incidence of TLR and MACE varied among quartiles (*P* = 0.011 and *P* = 0.002, respectively), with the lowest rates in the first quartile and the highest in the fourth (Table 1). No significant differences were observed in body surface area (BSA), Syntax score, or most laboratory parameters, as well as in categorical variables such as comorbidities and medication use.

**Table 1:** Baseline characteristics.

Variables	Q1 (*n* = 106)	Q2 (*n* = 105)	Q3 (*n* = 106)	Q4 (*n* = 106)	*P*
ePWV	10.47 (10.17,10.73)	11.39 (11.13,11.65)	12.32 (12.06,12.45)	13.58 (13.05,14.24)	**<**.**001**
Age, years	67.00 (66.00,69.00)	70.00 (67.00,71.00)	72.00 (69.00,75.00)	77.00 (74.00,80.75)	**<**.**001**
BSA, m2	1.66 (1.60,1.70)	1.65 (1.60,1.70)	1.65 (1.60,1.70)	1.64 (1.60,1.69)	0.128
Male, *n* (%)	70 (66.04)	63 (60.00)	64 (60.38)	67 (63.21)	0.784
Hypertension, *n* (%)	61 (57.55)	61 (58.10)	74 (69.81)	70 (66.04)	0.175
Diabetes, *n* (%)	28 (26.42)	34 (32.38)	37 (34.91)	22 (20.75)	0.100
Previous stroke, *n* (%)	14 (13.21)	20 (19.05)	14 (13.21)	19 (17.92)	0.521
Previous HF, (%)	14 (13.21)	6 (5.71)	9 (8.49)	15 (14.15)	0.146
AF, *n* (%)	6 (5.66)	6 (5.71)	7 (6.60)	12 (11.32)	0.333
Previous PCI, *n* (%)	29 (27.36)	18 (17.14)	31 (29.25)	30 (28.30)	0.153
Smoker, *n* (%)	26 (24.53)	18 (17.14)	21 (19.81)	24 (22.64)	0.573
WBC (*10^9^/L)	6.25 (5.23,7.38)	6.20 (5.20,7.00)	6.10 (5.30,7.20)	5.80 (5.03,6.60)	0.242
Mean Cell Volume (fl)	93.15 (90.53,95.70)	93.00 (90.70,96.20)	92.46 (91.00,95.35)	93.70 (92.03,95.70)	0.110
Red cell distribution width (%)	12.90 (12.60,13.28)	12.80 (12.40,13.20)	12.91 (12.50,13.20)	12.82 (12.43,13.20)	0.625
Hs-CRP (mg/L)	2.88 (1.38,7.91)	2.95 (1.45,7.10)	2.55 (0.90,6.36)	2.50 (0.93,6.22)	0.707
ALP (U/L)	72.00 (65.00,85.00)	76.00 (63.00,88.00)	78.98 (63.00,86.81)	76.32 (65.50,85.75)	0.544
Serum creatinine (umol/L)	66.00 (55.00,75.00)	64.00 (55.00,72.00)	65.86 (59.01,72.75)	67.06 (57.25,78.00)	0.316
Blood glucose (mmol/L)	5.55 (4.88,6.25)	5.61 (5.06,7.01)	5.68 (5.12,6.92)	5.35 (4.88,6.21)	0.141
TC (mmol/L)	3.84 (3.20,4.59)	4.12 (3.50,4.76)	4.14 (3.24,4.71)	4.01 (3.42,4.75)	0.360
TG (mmol/L)	1.40 (1.05,1.82)	1.38 (1.03,1.94)	1.34 (0.99,1.73)	1.35 (0.96,1.67)	0.667
HDL (mmol/L)	1.04 (0.87,1.19)	1.10 (0.90,1.23)	1.06 (0.92,1.17)	1.06 (0.93,1.21)	0.691
LDL (mmol/L)	2.17 (1.65,2.89)	2.42 (1.92,3.11)	2.46 (1.80,3.02)	2.30 (1.88,3.01)	0.439
Syntax score	13.50 (9.00,16.00)	14.00 (12.00,15.00)	13.00 (12.00,15.00)	14.00 (12.00,15.00)	0.775
Drug ballon diameter (mm)	2.50 (2.00,2.50)	2.00 (2.00,2.50)	2.50 (2.00,2.69)	2.50 (2.00,2.75)	0.327
Drug ballon length (mm)	20.00 (20.00,26.00)	20.00 (20.00,30.00)	20.00 (20.00,30.00)	20.00 (20.00,26.00)	0.804
Moderate calcification, *n* (%)	16 (15.09)	17 (16.19)	28 (26.42)	26 (24.53)	0.093
Cutting Balloon, *n* (%)	64 (60.38)	61 (58.10)	62 (58.49)	66 (62.26)	0.922
Aspirin, *n* (%)	102 (96.23)	103 (98.10)	101 (95.28)	99 (93.40)	0.416
P2Y12inhibitor, *n* (%)					0.837
Clopidogrel, *n* (%)	53 (50.00)	46 (43.81)	50 (47.17)	51 (48.11)	
Ticagrelor, *n* (%)	53 (50.00)	59 (56.19)	56 (52.83)	55 (51.89)	
Number of diseased vessels, *n* (%)					0.085
1	29 (27.36)	19 (18.10)	22 (20.75)	13 (12.26)	
2	30 (28.30)	45 (42.86)	40 (37.74)	47 (44.34)	
3	47 (44.34)	41 (39.05)	44 (41.51)	46 (43.40)	
TLR, *n* (%)	2 (1.89)	13 (12.38)	15 (14.15)	14 (13.21)	**0**.**011**
MACE, *n* (%)	3 (2.83)	13 (12.38)	18 (16.98)	20 (18.87)	**0**.**002**

HF, heart failure; MCV, mean cell volume; ALP, alkaline phosphatase; WBC, white blood cell count; RDW, red cell distribution width; TC, total cholesterol; TG, triglyceride; HDL-C, high-density lipoprotein cholesterol; LDL-C, low-density lipoprotein cholesterol; SCr, serum creatinine; ePWV, estimated pulse wave velocity; TLR, target lesion revascularization; MACE, major adverse cardiovascular events.

Bold values indicate statistical significance (*P* < 0.05).

### Variables associated with increased risk of Tlr were analyzed with Cox regression survival analysis

To prevent overfitting given the limited number of events, only variables showing significant associations (*P* < 0.05) in the univariate analysis were included in the multivariate models (Supplementary Tables S1 and S2). Cox regression analysis revealed an association between ePWV and TLR risk, whether analyzed as a continuous variable or categorized by quartiles. Each unit increase in ePWV correlated with heightened TLR risk across all models, exhibiting an adjusted HR of 1.46 (95% CI: 1.18∼1.79; *P* < 0.001) in the multivariate model. Quartile analysis revealed a pronounced dose-response relationship (P for trend < 0.05), with Q4 exhibiting the highest risk compared to Q1 (Table 2). Similarly associations emerged for MACE, findings across both crude and adjusted models (Table 2).

**Table 2A:** Association of ePWV and ePWV quantile with TLR.

Variables	Model 1		Model 2	
	HR (95%CI)	*P*	HR (95%CI)	*P*
ePWV	1.43 (1.16∼1.76)	**<.001**	1.46 (1.18∼1.79)	**<.001**
ePWV quantile				
Q1	1.00 (Reference)		1.00 (Reference)	
Q2	6.83 (1.54∼30.25)	**0.011**	7.38 (1.66∼32.83)	**0.009**
Q3	7.78 (1.78∼34.03)	**0.006**	9.02 (2.03∼40.17)	**0.004**
Q4	7.63 (1.74∼33.59)	**0.007**	9.00 (2.02∼40.03)	**0.004**
P for trend		**0.007**		**0.002**

HR, Hazard Ratio; CI, Confidence Interval.

Model 1: Crude.

Model 2: P2Y12inhibitor, Moderate calcification, Previous stent, Hs-CRP.

Bold values indicate statistical significance (*P* < 0.05).

**Table 2B:** Association of ePWV and ePWV quantile with MACE.

Variables	Model 1		Model 2	
	HR (95%CI)	*P*	HR (95%CI)	*P*
ePWV	1.51 (1.25∼1.82)	**<.001**	1.50 (1.25∼1.80)	**<.001**
ePWV quantile				
Q1	1.00 (Reference)		1.00 (Reference)	
Q2	4.55 (1.30∼15.98)	**0.018**	5.77 (1.63∼20.46)	**0.007**
Q3	6.26 (1.85∼21.27)	**0.003**	8.14 (2.36∼28.12)	**<.001**
Q4	7.28 (2.16∼24.49)	**0.001**	8.85 (2.60∼30.19)	**<.001**
P for trend		**<.001**		**<.001**

HR, Hazard Ratio; CI, Confidence Interval.

Model 1: Crude.

Model 2: P2Y12inhibitor, Previous stent, Syntax score, Drug ballon diameter, Hs-CRP.

Bold values indicate statistical significance (*P* < 0.05).

### Kaplan–Meier survival analysis by ePWV quartiles

**Figure 2:**
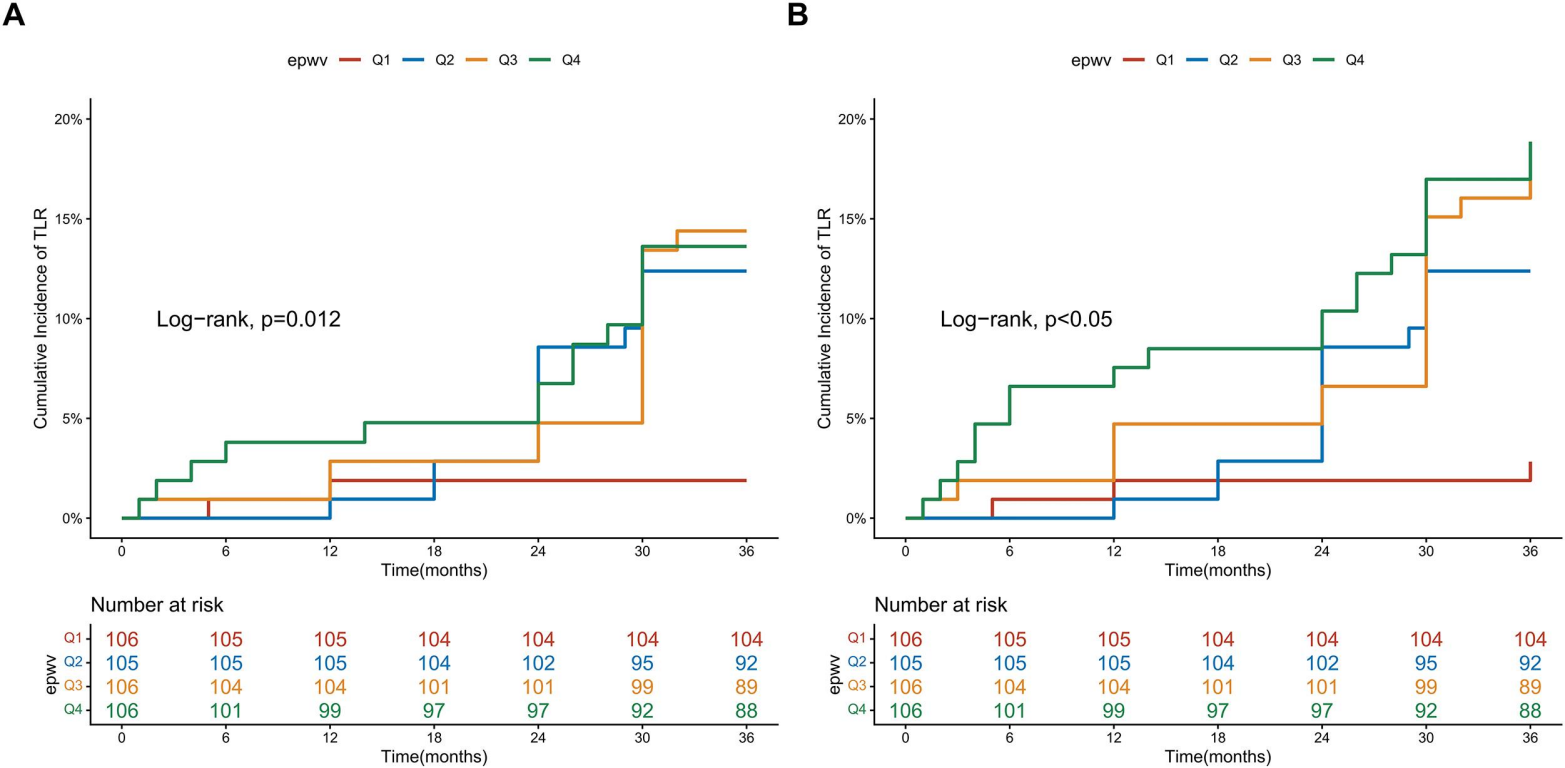
Kaplan–meier survival curves for clinical outcomes stratified by ePWV quartiles. **(A)** Kaplan–Meier estimates for freedom from TLR; **(B)** Kaplan–Meier estimates for freedom from MACE.

Kaplan–Meier analysis demonstrated differences in TLR-free survival across ePWV quartiles (log-rank test, *P* = 0.012) (Figure 2A). The Q1 group (lowest ePWV) exhibited the highest survival probability, whereas the Q4 group showed the lowest, with Q2 and Q3 showing intermediate risks. A similar graded pattern was observed for MACE (log-rank test, *P* < 0.05) (Figure 2B). The observed irregularities and overlapping of survival curves are likely attributable to the limited sample size and number of events in this single-center cohort.

### Establishing the optimal ePWV threshold for risk stratification

**Figure 3:**
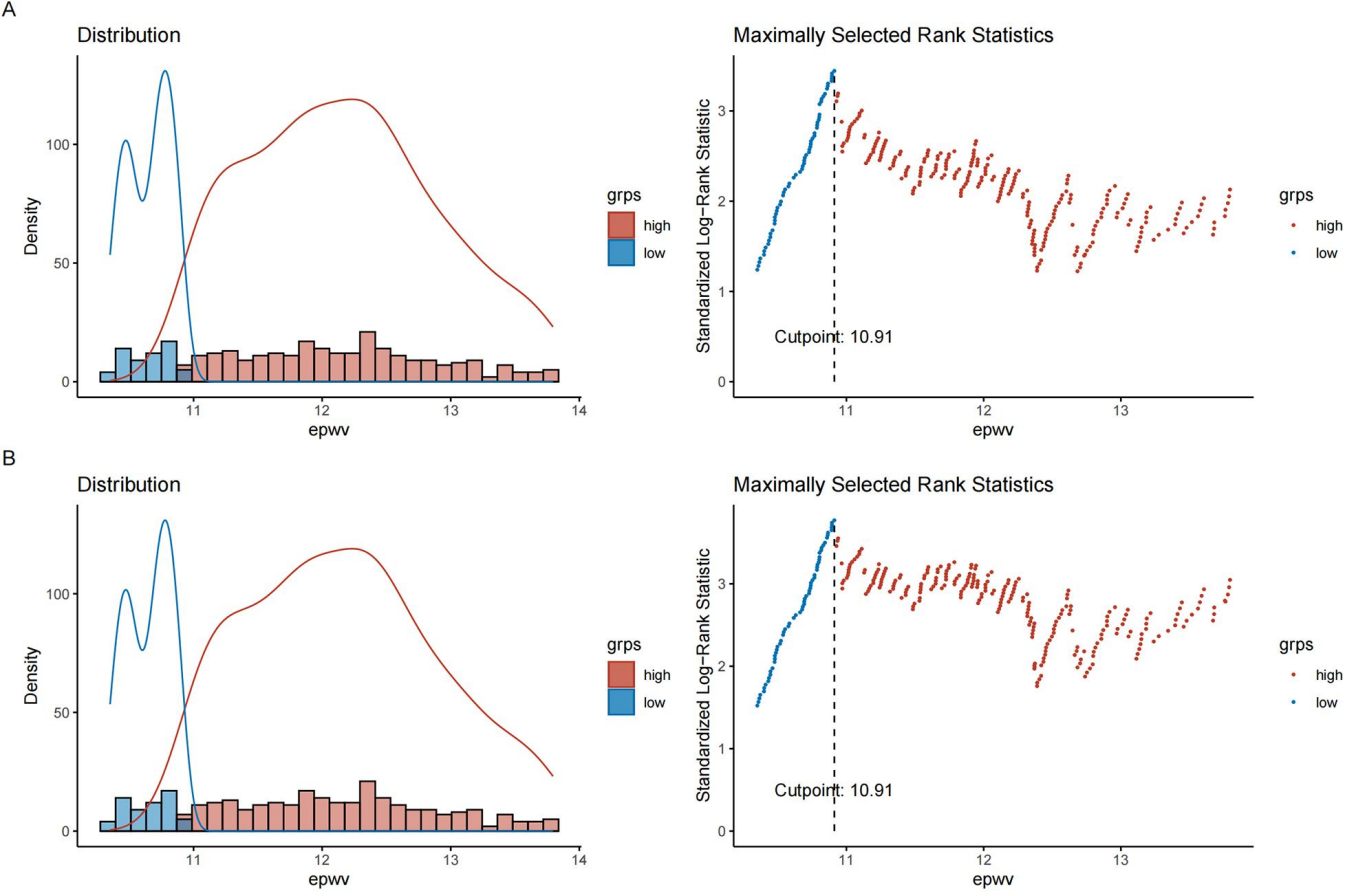
Identification of the optimal ePWV cutoff value using maximally selected rank statistics. **(A)** The optimal cutoff for predicting TLR; **(B)** The optimal cutoff for predicting MACE. The vertical dashed line indicates the determined optimal cutoff value of 10.91 m/s, which corresponds to the maximum standardized log-rank statistic.

**Figure 4:**
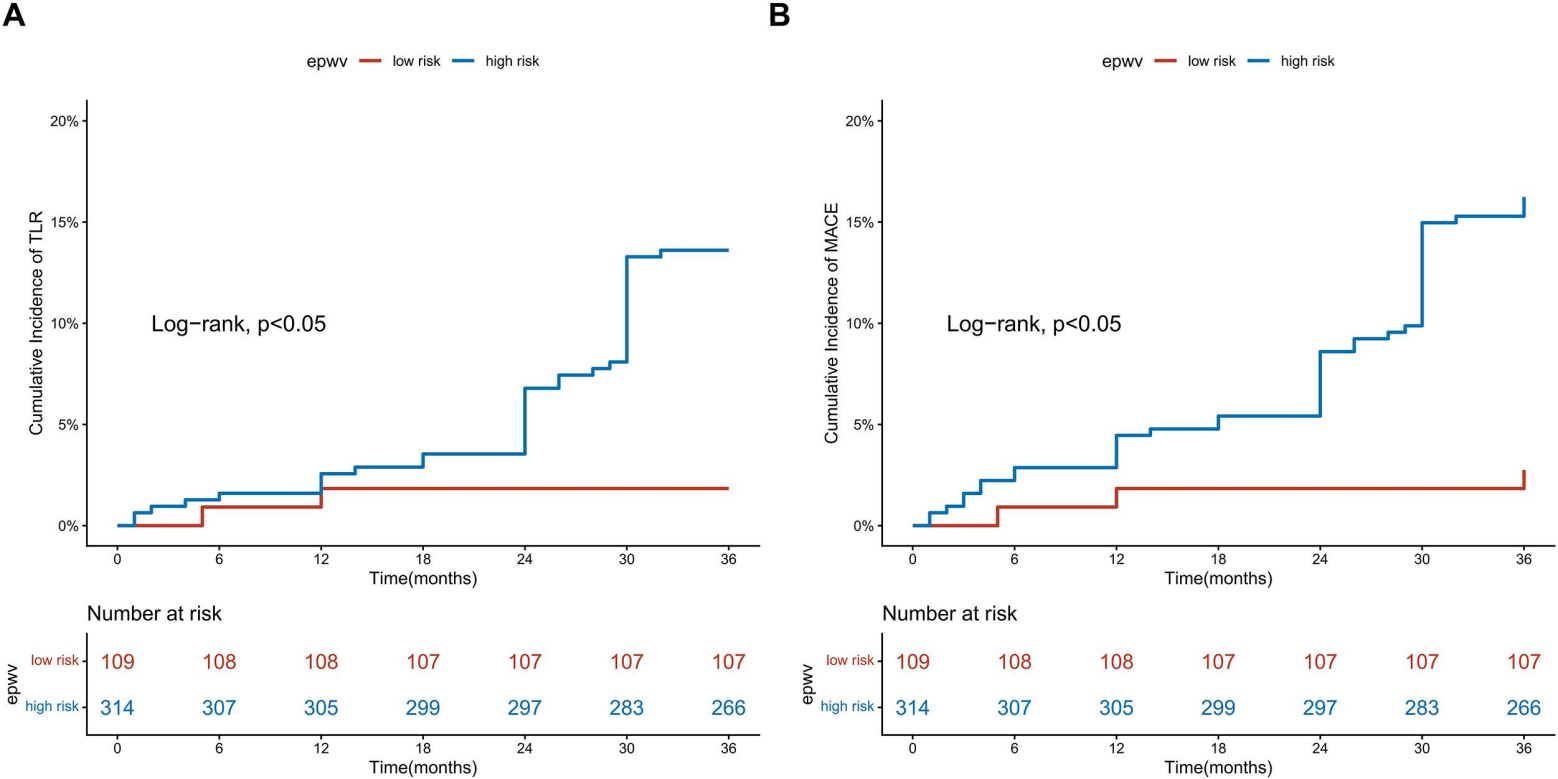
Kaplan–meier survival analysis stratified by the optimal ePWV cutoff value. **(A)** Freedom from TLR; **(B)** Freedom from MACE. Patients were stratified into low-risk (<10.91 m/s) and high-risk (≥10.91 m/s) groups.

Maximally selected rank statistics identified an optimal ePWV cutoff of 10.91 m/s for predicting TLR and MACE risk, corresponding to the maximum standardized log-rank statistic. Kaplan–Meier curves demonstrated a difference in outcomes between the high-risk (ePWV ≥ 10.91 m/s) and low-risk (ePWV < 10.91 m/s) groups (log-rank test, *P* < 0.05) (Figure 3). Throughout the follow-up period, the high-risk group showed lower rates of TLR-free and MACE-free survival compared to the low-risk group (Figure 4).

### Subgroup analysis

**Figure 5:**
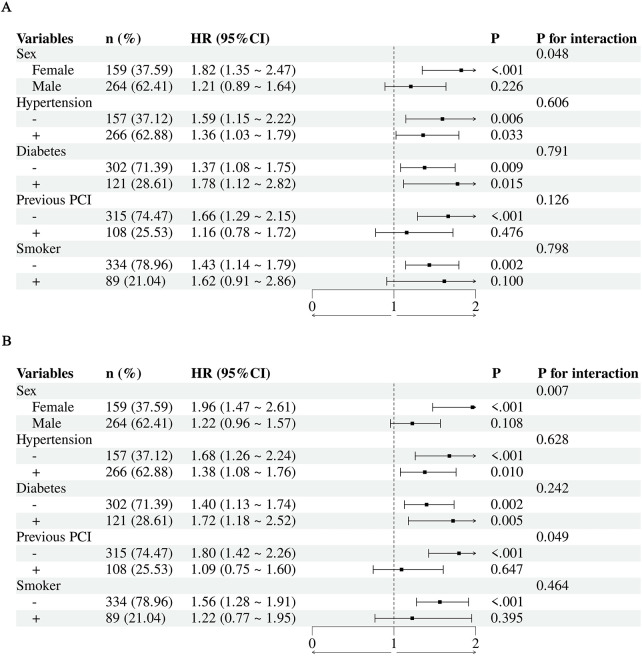
Forest plots of subgroup analyses. **(A)** HR for TLR across different subgroups; **(B)** HR for MACE across different subgroups.

This subgroup analysis of 423 patients explored factors influencing target lesion revascularization (TLR) and major adverse cardiovascular events (MACE). Overall, the analysis demonstrated an elevated risk in the total population (HR 1.46, 95% CI 1.18–1.79, *P* < 0.001) (Figure 5A). Notably, interactions by sex were observed for both TLR and MACE (*P* < 0.05) (Figures 5A,B). The association was consistently more pronounced in females compared to males for both endpoints, suggesting that elderly female patients may be particularly vulnerable to the risks associated with elevated ePWV. In contrast, no significant interactions were observed across other subgroups stratified by hypertension, diabetes, smoking history, or prior PCI.

### Sensitivity analyses

To verify the reliability of our findings, we performed comprehensive sensitivity analyses. We evaluated the association between ePWV and clinical outcomes using different adjustment models. The adjusted HR for ePWV per 1 m/s increase remained robust, at 2.58 for TLR and 2.43 for MACE (Supplementary Table S3). Additionally, analyses excluding participants with extreme ePWV values (top and bottom 2%) yielded consistent findings, with HRs of 1.35 for TLR and 1.47 for MACE (Supplementary Table S4).

## Discussion

This study demonstrates that elevated estimated pulse wave velocity (ePWV) was associated with a higher risk of target lesion revascularization (TLR) and major adverse cardiovascular events (MACE) in elderly patients with coronary heart disease after drug-coated balloon (DCB) angioplasty.

DCB angioplasty represents a “stent-free” interventional approach, delivering potent antiproliferative drugs directly to inhibit neointimal hyperplasia while eliminating the need for permanent metallic implants. Compared with drug-eluting stents (DES), DCB significantly simplifies the procedural technique, reduces contrast volume requirements, and shortens dual antiplatelet therapy (DAPT) duration—rendering it particularly advantageous for patients at high bleeding risk (20). Clinical trials confirm DCB achieves comparable clinical efficacy to DES in treating *de novo* lesions, supporting its broader implementation (21). Nonetheless, the occurrence rates of TLR and MACE following DCB intervention remain a significant clinical concern, particularly within complex anatomies or high-risk patient subgroups, potentially limiting its widespread adoption.

Our investigation reveals that ePWV exhibits an association with heightened risks of TLR and MACE in patients undergoing DCB treatment. The pronounced dose-response relationship across ePWV quartiles, coupled with an elevated hazard in the highest quartile. Notably, the wide 95% CI indicates substantial imprecision in the point estimate of HR due to the limited number of events, and the point estimates are too imprecise to be taken literally. Furthermore, the empirically derived ePWV cutoff of 10.91 m/s provides a clinically actionable risk stratification threshold, aiding identification of patients with substantially elevated TLR risk during long-term follow-up. Importantly, this cutoff is exploratory as it was established in the current cohort, and it requires external validation in independent populations to confirm robustness and generalizable utility.

These findings corroborate with prior research underscoring the prognostic importance of arterial stiffness in cardiovascular disease. Our results align with previous studies establishing ePWV as a predictor of adverse outcomes in CAD patients (10, 22). In a landmark study encompassing 12,792 U.S. adults, each 1 m/s increase in ePWV demonstrated a association with a 15% higher all-cause mortality risk (HR 1.15, 95% CI 1.10–1.20) following comprehensive multivariable adjustment. Individuals within the highest ePWV quartile faced a more than twofold surge in mortality risk (HR 2.24, 95% CI 1.72–2.92), reinforcing ePWV's status as a mortality predictor (23). Moreover, a male cohort study further solidified this relationship, revealing that elevated ePWV predicted heightened cardiovascular risk. Each 1 m/s increase was linked to a 13% rise in cardiovascular events (HR 1.13, 95% CI 1.06–1.21), even after accounting for conventional risk factors (24).

The specific contributions of ePWV in clarifying the mechanisms on outcomes following DCB treatment remain unclear. The following potential explanations are proposed. Long-term uncontrolled hypertension, a primary driver of arterial stiffness, accelerates atherosclerosis and restenosis, potentially mediating ePWV's predictive value. Elevated arterial stiffness can harm the delicate microstructure of blood vessels, triggering microvascular dysfunction, impairing arterial buffering capacity, and increasing the risk of vascular disease (25), thereby creating a vulnerable vascular environment. Crucially, elevated ePWV correlates with more complex coronary lesions, impaired vascular healing, and adverse hemodynamics (26, 27). These factors may translate into higher restenosis rates and increased adverse events post-DCB treatment. Beyond this, frailty, prevalent in elderly populations, correlates with arterial stiffness and predicts poor post-intervention outcomes via age-related declines in physiological reserve (28). While direct clinical evidence within CAD is currently limited, future research should rigorously evaluate ePWV alongside established prognostic markers. ePWV serves as a reflection of systemic vascular dysfunction and may enhance outcome prediction following endovascular therapies like DCBs (8).

Notably, sex stratified analyses revealed a pronounced interaction among elderly female patients, who exhibited an elevated risk. This finding underscores the important role of sex specific vascular pathophysiology in clinical outcomes. Postmenopausal estrogen loss accelerates arterial stiffness through endothelial dysfunction, oxidative stress, and calcification. Inherent sex-based arterial differences, including smaller vessel diameter and reduced remodeling, further amplify hemodynamic vulnerability and compromise post-intervention healing (29). However, this sex-related finding preliminary and exploratory.

The prognostic value of ePWV in elderly patients undergoing DCB therapy should be interpreted in light of evolving evidence on DCB technology. The REC-CAGEFREE I trial (30), which showed paclitaxel-coated balloons (PCBs) to be inferior to sirolimus-eluting stents, reflects outcomes specific to one DCB platform in a particular clinical setting. Importantly, DCB platforms are heterogeneous. Emerging evidence indicates sirolimus-coated balloons (SCBs) may offer advantages or noninferiority over PCBs, including a lower binary restenosis rate in bifurcation lesions (31) and demonstrated noninferiority in late lumen loss for treating in-stent restenosis (32). These lesion types are common among elderly patients. These differences likely stem from variations in drug pharmacokinetics and coating technology. Future studies stratifying outcomes by both vascular stiffness (ePWV) and DCB platform are needed to refine patient selection for this high-risk elderly population.

This single-center retrospective study has limitations. First, selection bias cannot be ruled out due to strict exclusion criteria and the single-center design, potentially limiting generalizability. Second, unmeasured confounders, such as medication adherence or socioeconomic status, may have influenced outcomes. Third, statistical stability was limited by insufficient event counts in subgroups and multicollinearity when simultaneously adjusting for age and blood pressure (Supplementary Tables S5 & S6), which likely contributed to inflated hazard ratios. Fourth, the ePWV formula lacks specific validation in elderly PCI patients. Finally, the 3-year follow-up may miss late events, and the findings indicate correlation rather than causality.

## Conclusion

Elevated ePWV correlates with elevated risks of TLR and MACE in elderly coronary heart disease patients following drug-coated balloon angioplasty. The exploratory cutoff value of 10.91 m/s represents a promising tool for risk assessment; however, its clinical application requires additional validation.

## Data Availability

The raw data supporting the conclusions of this article will be made available by the authors, without undue reservation.
