# Supplementary material for: Association of estimated pulse wave velocity with outcomes following drug-coated balloon therapy in elderly coronary artery disease patients
Source: Front Cardiovasc Med. 2026 Feb 13;13:1706461. doi: 10.3389/fcvm.2026.1706461 (PMC12945768; doi:10.3389/fcvm.2026.1706461)
Supplement: Supplementary file 1 [file Datasheet1.docx]

| **Supplementary File 1:** Univariate Cox Proportional Hazards Model for Target Lesion TLR | | | | | |
| --- | --- | --- | --- | --- | --- |
| Variables | β | S.E | Z | *P* | HR (95%CI) |
|  |  |  |  |  |  |
| P2Y12inhibitor |  |  |  |  |  |
| Clopidogrel |  |  |  |  | 1.00 (Reference) |
| Ticagrelor | -0.79 | 0.32 | -2.48 | **0.013** | 0.45 (0.24 ~ 0.85) |
| Sex |  |  |  |  |  |
| 1 |  |  |  |  | 1.00 (Reference) |
| 2 | 0.35 | 0.30 | 1.16 | 0.247 | 1.42 (0.78 ~ 2.57) |
| Hypertension |  |  |  |  |  |
| 0 |  |  |  |  | 1.00 (Reference) |
| 1 | -0.18 | 0.31 | -0.58 | 0.560 | 0.84 (0.46 ~ 1.53) |
| Diabetes |  |  |  |  |  |
| 0 |  |  |  |  | 1.00 (Reference) |
| 1 | 0.17 | 0.32 | 0.53 | 0.596 | 1.19 (0.63 ~ 2.24) |
| Previous stroke |  |  |  |  |  |
| 0 |  |  |  |  | 1.00 (Reference) |
| 1 | 0.36 | 0.37 | 0.96 | 0.338 | 1.43 (0.69 ~ 2.98) |
| Previous HF |  |  |  |  |  |
| 0 |  |  |  |  | 1.00 (Reference) |
| 1 | -1.65 | 1.01 | -1.63 | 0.103 | 0.19 (0.03 ~ 1.40) |
| AF |  |  |  |  |  |
| 0 |  |  |  |  | 1.00 (Reference) |
| 1 | -0.53 | 0.72 | -0.74 | 0.460 | 0.59 (0.14 ~ 2.42) |
| Previous PCI |  |  |  |  |  |
| 0 |  |  |  |  | 1.00 (Reference) |
| 1 | 0.21 | 0.33 | 0.64 | 0.523 | 1.23 (0.65 ~ 2.36) |
| Smoker |  |  |  |  |  |
| 0 |  |  |  |  | 1.00 (Reference) |
| 1 | -0.19 | 0.39 | -0.47 | 0.635 | 0.83 (0.39 ~ 1.79) |
| Aspirin |  |  |  |  |  |
| 0 |  |  |  |  | 1.00 (Reference) |
| 1 | 0.60 | 1.01 | 0.59 | 0.553 | 1.82 (0.25 ~ 13.23) |
| Number of diseased vessels |  |  |  |  |  |
| 1 |  |  |  |  | 1.00 (Reference) |
| 2 | 0.45 | 0.44 | 1.04 | 0.298 | 1.58 (0.67 ~ 3.71) |
| 3 | 0.06 | 0.45 | 0.14 | 0.891 | 1.06 (0.44 ~ 2.59) |
| Moderate calcification |  |  |  |  |  |
| 0 |  |  |  |  | 1.00 (Reference) |
| 1 | 0.71 | 0.32 | 2.22 | **0.027** | 2.02 (1.09 ~ 3.78) |
| Cutting Balloon |  |  |  |  |  |
| 0 |  |  |  |  | 1.00 (Reference) |
| 1 | -0.23 | 0.30 | -0.76 | 0.445 | 0.79 (0.44 ~ 1.44) |
| Previous stent |  |  |  |  |  |
| 0 |  |  |  |  | 1.00 (Reference) |
| 1 | -0.73 | 0.35 | -2.09 | **0.036** | 0.48 (0.24 ~ 0.95) |
| Age | 0.03 | 0.03 | 1.13 | 0.256 | 1.03 (0.98 ~ 1.09) |
| BSA | -1.78 | 1.96 | -0.91 | 0.365 | 0.17 (0.00 ~ 7.90) |
| Syntay score | 0.07 | 0.04 | 1.80 | 0.071 | 1.07 (0.99 ~ 1.15) |
| Drug ballon diameter | 0.59 | 0.31 | 1.94 | 0.052 | 1.81 (1.00 ~ 3.29) |
| Drug ballon length | -0.02 | 0.03 | -0.67 | 0.505 | 0.98 (0.93 ~ 1.03) |
| WBC | 0.10 | 0.07 | 1.45 | 0.146 | 1.11 (0.96 ~ 1.28) |
| LY | -0.03 | 0.02 | -1.37 | 0.172 | 0.97 (0.94 ~ 1.01) |
| Mean Cell Volume | -0.04 | 0.03 | -1.61 | 0.107 | 0.96 (0.91 ~ 1.01) |
| RDW | 0.10 | 0.15 | 0.68 | 0.499 | 1.10 (0.83 ~ 1.47) |
| CRP | 0.02 | 0.00 | 3.29 | **0.001** | 1.02 (1.01 ~ 1.03) |
| ALP | -0.00 | 0.01 | -0.22 | 0.824 | 1.00 (0.98 ~ 1.01) |
| AB | 0.00 | 0.03 | 0.17 | 0.861 | 1.00 (0.95 ~ 1.06) |
| Serum creatinine | -0.01 | 0.01 | -0.92 | 0.357 | 0.99 (0.97 ~ 1.01) |
| Blood glucose | 0.04 | 0.07 | 0.59 | 0.556 | 1.04 (0.91 ~ 1.19) |
| TC | -0.04 | 0.11 | -0.40 | 0.692 | 0.96 (0.77 ~ 1.19) |
| TG | 0.08 | 0.15 | 0.53 | 0.596 | 1.08 (0.81 ~ 1.46) |
| HDL | -0.93 | 0.65 | -1.42 | 0.154 | 0.39 (0.11 ~ 1.42) |
| LDL | -0.10 | 0.18 | -0.53 | 0.599 | 0.91 (0.63 ~ 1.30) |
| MAP | 0.04 | 0.01 | 3.45 | **<.001** | 1.04 (1.02 ~ 1.07) |
| Epwv | 0.36 | 0.11 | 3.34 | **<.001** | 1.43 (1.16 ~ 1.76) |
| Epwv Quantile |  |  |  |  |  |
| 1 |  |  |  |  | 1.00 (Reference) |
| 2 | 1.92 | 0.76 | 2.53 | **0.011** | 6.83 (1.54 ~ 30.25) |
| 3 | 2.05 | 0.75 | 2.73 | **0.006** | 7.78 (1.78 ~ 34.03) |
| 4 | 2.03 | 0.76 | 2.69 | **0.007** | 7.63 (1.74 ~ 33.59) |
| systolic blood pressure | 0.03 | 0.01 | 3.78 | **<.001** | 1.03 (1.01 ~ 1.05) |
| diastolic blood pressure | 0.03 | 0.01 | 2.35 | **0.019** | 1.03 (1.01 ~ 1.06) |
| HR: Hazard Ratio, CI: Confidence Interval | | | | | |

| **Supplementary File 2：**Univariate Cox proportional hazards model analysis for MACE. | | | | | |
| --- | --- | --- | --- | --- | --- |
| Variables | β | S.E | Z | *P* | HR (95%CI) |
|  |  |  |  |  |  |
| P2Y12inhibitor |  |  |  |  |  |
| Clopidogrel |  |  |  |  | 1.00 (Reference) |
| Ticagrelor | -0.58 | 0.28 | -2.09 | **0.037** | 0.56 (0.32 ~ 0.97) |
| Sex |  |  |  |  |  |
| 1 |  |  |  |  | 1.00 (Reference) |
| 2 | 0.24 | 0.28 | 0.86 | 0.391 | 1.27 (0.74 ~ 2.17) |
| Hypertension |  |  |  |  |  |
| 0 |  |  |  |  | 1.00 (Reference) |
| 1 | -0.32 | 0.27 | -1.18 | 0.238 | 0.72 (0.42 ~ 1.24) |
| Diabetes |  |  |  |  |  |
| 0 |  |  |  |  | 1.00 (Reference) |
| 1 | 0.16 | 0.29 | 0.53 | 0.594 | 1.17 (0.66 ~ 2.08) |
| Previous stroke |  |  |  |  |  |
| 0 |  |  |  |  | 1.00 (Reference) |
| 1 | 0.35 | 0.34 | 1.04 | 0.300 | 1.42 (0.73 ~ 2.75) |
| Previous HF |  |  |  |  |  |
| 0 |  |  |  |  | 1.00 (Reference) |
| 1 | -1.86 | 1.01 | -1.84 | 0.066 | 0.16 (0.02 ~ 1.13) |
| AF |  |  |  |  |  |
| 0 |  |  |  |  | 1.00 (Reference) |
| 1 | -0.02 | 0.52 | -0.03 | 0.976 | 0.98 (0.36 ~ 2.73) |
| Previous PCI |  |  |  |  |  |
| 0 |  |  |  |  | 1.00 (Reference) |
| 1 | 0.03 | 0.31 | 0.09 | 0.925 | 1.03 (0.56 ~ 1.89) |
| Smoker |  |  |  |  |  |
| 0 |  |  |  |  | 1.00 (Reference) |
| 1 | 0.07 | 0.33 | 0.20 | 0.841 | 1.07 (0.56 ~ 2.03) |
| Aspirin |  |  |  |  |  |
| 0 |  |  |  |  | 1.00 (Reference) |
| 1 | -0.32 | 0.59 | -0.54 | 0.586 | 0.72 (0.23 ~ 2.32) |
| Number of diseased vessels |  |  |  |  |  |
| 1 |  |  |  |  | 1.00 (Reference) |
| 2 | 0.46 | 0.38 | 1.19 | 0.236 | 1.58 (0.74 ~ 3.36) |
| 3 | -0.07 | 0.41 | -0.17 | 0.865 | 0.93 (0.42 ~ 2.08) |
| Moderate calcification |  |  |  |  |  |
| 0 |  |  |  |  | 1.00 (Reference) |
| 1 | 0.41 | 0.30 | 1.36 | 0.174 | 1.51 (0.83 ~ 2.74) |
| Cutting Balloon |  |  |  |  |  |
| 0 |  |  |  |  | 1.00 (Reference) |
| 1 | -0.11 | 0.28 | -0.42 | 0.677 | 0.89 (0.52 ~ 1.53) |
| Previous stent |  |  |  |  |  |
| 0 |  |  |  |  | 1.00 (Reference) |
| 1 | -0.78 | 0.32 | -2.44 | **0.015** | 0.46 (0.25 ~ 0.86) |
| Age | 0.06 | 0.02 | 2.28 | **0.022** | 1.06 (1.01 ~ 1.11) |
| BSA | 0.09 | 1.79 | 0.05 | 0.961 | 1.09 (0.03 ~ 36.16) |
| Syntax score | 0.07 | 0.03 | 2.07 | **0.038** | 1.07 (1.01 ~ 1.15) |
| Drug ballon diameter | 0.71 | 0.27 | 2.62 | **0.009** | 2.03 (1.19 ~ 3.44) |
| Drug ballon length | 0.01 | 0.02 | 0.30 | 0.764 | 1.01 (0.96 ~ 1.05) |
| WBC | 0.06 | 0.07 | 0.82 | 0.414 | 1.06 (0.92 ~ 1.22) |
| LY | -0.02 | 0.02 | -1.31 | 0.191 | 0.98 (0.95 ~ 1.01) |
| MCV | -0.04 | 0.03 | -1.45 | 0.147 | 0.96 (0.92 ~ 1.01) |
| RDW | 0.02 | 0.15 | 0.15 | 0.880 | 1.02 (0.76 ~ 1.37) |
| CRP | 0.01 | 0.00 | 2.71 | **0.007** | 1.01 (1.01 ~ 1.02) |
| ALP | -0.00 | 0.01 | -0.13 | 0.899 | 1.00 (0.98 ~ 1.01) |
| AB | -0.01 | 0.03 | -0.37 | 0.710 | 0.99 (0.94 ~ 1.04) |
| SCR | -0.00 | 0.01 | -0.21 | 0.833 | 1.00 (0.98 ~ 1.01) |
| GLU | 0.06 | 0.06 | 1.07 | 0.284 | 1.06 (0.95 ~ 1.18) |
| TC | -0.02 | 0.08 | -0.27 | 0.788 | 0.98 (0.83 ~ 1.15) |
| TG | -0.01 | 0.16 | -0.04 | 0.970 | 0.99 (0.73 ~ 1.36) |
| HDL | -0.36 | 0.56 | -0.64 | 0.521 | 0.70 (0.23 ~ 2.10) |
| LDL | -0.02 | 0.16 | -0.10 | 0.921 | 0.98 (0.72 ~ 1.35) |
| MAP | 0.04 | 0.01 | 3.51 | **<.001** | 1.04 (1.02 ~ 1.06) |
| Epwv | 0.41 | 0.10 | 4.31 | **<.001** | 1.51 (1.25 ~ 1.82) |
| Epwv Quantile |  |  |  |  |  |
| 1 |  |  |  |  | 1.00 (Reference) |
| 2 | 1.52 | 0.64 | 2.37 | **0.018** | 4.55 (1.30 ~ 15.98) |
| 3 | 1.83 | 0.62 | 2.94 | **0.003** | 6.26 (1.85 ~ 21.27) |
| 4 | 1.98 | 0.62 | 3.21 | **0.001** | 7.28 (2.16 ~ 24.49) |
| systolic blood pressure | 0.03 | 0.01 | 3.80 | **<.001** | 1.03 (1.01 ~ 1.04) |
| diastolic blood pressure | 0.03 | 0.01 | 2.45 | **0.014** | 1.03 (1.01 ~ 1.05) |
| HR: Hazard Ratio, CI: Confidence Interval | | | | | |

| **Supplementary File 3**: Association of epwv and epwv quantile with TLR. | | | | | | | | |
| --- | --- | --- | --- | --- | --- | --- | --- | --- |
| **Variables** | **Model1** | |  | **Model2** | |  | **Model3** | |
|  | **HR (95%CI)** | ***P*** |  | **HR (95%CI)** | ***P*** |  | **HR (95%CI)** | ***P*** |
| epwv | 1.43 (1.16 ~ 1.76) | **<.001** |  | 2.11 (1.49 ~ 2.98) | **<.001** |  | 2.58 (1.75 ~ 3.82) | **<.001** |
| epwv quantile |  |  |  |  |  |  |  |  |
| Q1 | 1.00 (Reference) |  |  | 1.00 (Reference) |  |  | 1.00 (Reference) |  |
| Q2 | 6.83 (1.54 ~ 30.25) | **0.011** |  | 6.72 (1.50 ~ 30.16) | **0.013** |  | 10.22 (2.05 ~ 51.03) | **0.005** |
| Q3 | 7.78 (1.78 ~ 34.03) | **0.006** |  | 9.44 (2.05 ~ 43.47) | **0.004** |  | 17.16 (3.28 ~ 89.81) | **<.001** |
| Q4 | 7.63 (1.74 ~ 33.59) | **0.007** |  | 12.08 (2.31 ~ 63.13) | **0.003** |  | 28.72 (4.66 ~ 177.03) | **<.001** |
|  | | | | | | | | |
| Association of epwv and epwv quantile with MACE. | | | | | | | | |
| **Variables** | **Model1** | |  | **Model2** | |  | **Model3** | |
|  | **HR (95%CI)** | ***P*** |  | **HR (95%CI)** | ***P*** |  | **HR (95%CI)** | ***P*** |
| epwv | 1.51 (1.25 ~ 1.82) | **<.001** |  | 2.07 (1.52 ~ 2.84) | **<.001** |  | 2.43 (1.72 ~ 3.44) | **<.001** |
| epwv quantile |  |  |  |  |  |  |  |  |
| Q1 | 1.00 (Reference) |  |  | 1.00 (Reference) |  |  | 1.00 (Reference) |  |
| Q2 | 4.55 (1.30 ~ 15.98) | **0.018** |  | 4.28 (1.21 ~ 15.19) | **0.025** |  | 6.03 (1.60 ~ 22.68) | **0.008** |
| Q3 | 6.26 (1.85 ~ 21.27) | **0.003** |  | 7.23 (2.03 ~ 25.78) | **0.002** |  | 13.13 (3.36 ~ 51.36) | **<.001** |
| Q4 | 7.28 (2.16 ~ 24.49) | **0.001** |  | 9.96 (2.49 ~ 39.84) | **0.001** |  | 21.55 (4.86 ~ 95.57) | **<.001** |
| Note:HR: Hazard Ratio, CI: Confidence Interval  Model 1: Crude  Model 2: Adjust:Sex, Hypertension, Diabetes, Stroke, Heart Failure, Atrial Fibrillation, Previous Percutaneous Coronary Intervention (PCI), Smoking History, Age, Body Surface Area (BSA)  Model 3: Adjust: Sex, Hypertension, Diabetes, Stroke, Heart Failure, Atrial Fibrillation, Previous Percutaneous Coronary Intervention (PCI), Smoking, Aspirin Use, Number of Diseased Vessels, Calcification, Cutting Balloon Use, Age, Body Surface Area (BSA), SYNTAX Score, Drug-Coated Balloon Diameter, Drug-Coated Balloon Length, White Blood Cell Count (WBC), Mean Corpuscular Volume (MCV), Red Cell Distribution Width (RDW), C-Reactive Protein (CRP), Alkaline Phosphatase (ALP), Albumin (AB), Serum Creatinine (SCR), Glucose (GLU), Cholesterol, Triglycerides, High-Density Lipoprotein (HDL), Low-Density Lipoprotein (LDL) | | | | | | | | |

| **Supplementary File 4.** Association of ePWV and ePWV quartiles with TLR excluding participants with extreme ePWV values (top and bottom 2%). | | | | |
| --- | --- | --- | --- | --- |
| **Variables** | **Model1** | | **Model2** | |
|  | **HR (95%CI)** | ***P*** | **HR (95%CI)** | ***P*** |
| epwv | 1.30 (1.01 ~ 1.67) | **0.044** | 1.35 (1.04 ~ 1.74) | **0.021** |
| epwv quantile |  |  |  |  |
| Q1 | 1.00 (Reference) |  | 1.00 (Reference) |  |
| Q2 | 6.33 (1.43 ~ 28.07) | **0.015** | 6.97 (1.57 ~ 31.00) | **0.011** |
| Q3 | 7.27 (1.66 ~ 31.79) | **0.008** | 8.70 (1.96 ~ 38.70) | **0.004** |
| Q4 | 5.38 (1.18 ~ 24.57) | **0.030** | 6.40 (1.39 ~ 29.46) | **0.017** |
| P for trend |  | **0.047** |  | **0.021** |
| Note: HR: Hazard Ratio, CI: Confidence Interval  Model 1: Crude  Model 2: P2Y12inhibitor, Moderate calcification, Previous stent, Hs-CRP | | | | |
|  | | | | |
| Association of ePWV and ePWV quartiles with MACE excluding participants with extreme ePWV values (top and bottom 2%). | | | | |
| **Variables** | **Model1** | | **Model2** | |
|  | **HR (95%CI)** | ***P*** | **HR (95%CI)** | ***P*** |
| epwv | 1.45 (1.16 ~ 1.82) | **0.001** | 1.47 (1.17 ~ 1.84) | **0.001** |
| epwv quantile |  |  |  |  |
| Q1 | 1.00 (Reference) |  | 1.00 (Reference) |  |
| Q2 | 4.21 (1.20 ~ 14.78) | **0.025** | 4.88 (1.38 ~ 17.22) | **0.014** |
| Q3 | 5.83 (1.72 ~ 19.81) | **0.005** | 6.91 (2.02 ~ 23.59) | **0.002** |
| Q4 | 5.74 (1.67 ~ 19.69) | **0.005** | 6.45 (1.87 ~ 22.26) | **0.003** |
| P for trend |  | **0.003** |  | **0.002** |
| Note: HR: Hazard Ratio, CI: Confidence Interval  Model 1: Crude  Model 2: P2Y12inhibitor, Previous stent, Syntax score, Drug ballon diameter, Hs-CRP | | | | |
